# Association of systemic inflammation response index with latent tuberculosis infection and all-cause mortality: a cohort study from NHANES 2011-2012

**DOI:** 10.3389/fimmu.2025.1538132

**Published:** 2025-02-19

**Authors:** Lin Wang, Yi Kuang, Yan Zeng, Zhihui Wan, Sha Yang, Renliang Li

**Affiliations:** ^1^ Infectious Disease Department, Jiangxi Province Hospital of Integrated Traditional Chinese and Western Medicine, Nanchang, Jiangxi, China; ^2^ Graduate School, Jiangxi University of Chinese Medicine, Nanchang, Jiangxi, China

**Keywords:** systemic inflammation response index, all-cause mortality, latent tuberculosis infection, inflammatory biomarker, NHANES

## Abstract

**Background:**

The Systemic Inflammatory Response Index (SIRI) is a promising inflammatory marker; however, the relationship between SIRI and latent tuberculosis infection (LTBI), as well as its association with mortality rates, remains unclear. The present study aimed to explore the associations of the SIRI with LTBI and all-cause mortality.

**Methods:**

We conducted a prospective cohort study using data from National Health and Nutrition Examination Survey (NHANES) cycles from 2011 to 2012. We explored the association between SIRI and LTBI prevalence using Multiple logistic regression models. We used Multivariate Cox proportional hazards model to analyze the association between SIRI and all-cause mortality. In addition, Kaplan-Meier curves, restricted cubic splines (RCS), stratified analysis, and interaction tests were performed.

**Results:**

The study included a total of 4,983 adults who participated in NHANES 2011-2012. The mean follow-up period was 92.35 ± 16.82 months, and there were 525 deaths, representing a mortality rate of 10.54%. The occurrence of LTBI is significantly associated with low SIRI levels(OR=0.76, 95% CI: 0.66-0.89), after adjusting for confounders. Among LTBI patients, elevated SIRI levels are significantly correlated with an increased risk of all-cause mortality (adjusted HR = 1.48, 95% CI: 1.01-2.15). RCS revealed a linear relationship between SIRI and all-cause mortality in patients with LTBI (*P* =0.059[overall] and *P* = 0.391 [Nonlinear]). Furthermore, within the LTBI population, the area under the curve (AUC) of SIRI for all-cause mortality are 0.731 (1-year), 0.640 (3-year), and 0.634 (5-year).

**Conclusion:**

The findings of this study indicate that elevated SIRI levels are independently associated with an increased risk of all-cause mortality in patients with LTBI. Notably, SIRI was significantly inversely associated with the incidence of LTBI. Therefore, SIRI may serve as an effective tool for risk stratification in adults with LTBI in the United States. Further research is needed to elucidate the underlying mechanisms and to explore any therapeutic implications of these findings.

## 1 Introduction

Infection with Mycobacterium tuberculosis (M.tb) is traditionally thought to lead to three different outcomes in the human body: the eradication of M.tb, the development of active tuberculosis (TB), and the establishment of latent tuberculosis infection (LTBI). However, recent work by Coussens et al. (1) in *The Lancet Respiratory Medicine* proposes a more nuanced classification of outcomes following M.tb infection, highlighting the complexity of the disease spectrum (1). The term LTBI signifies the presence of M.tb within the host, albeit in a “dormant state” without any corresponding clinical manifestations. Once the immune system is compromised, M.tb has the opportunity to proliferate and cause disease (2). It is imperative not to underestimate the significance of LTBI, as approximately one-fifth of the global population is affected by it (3). Although LTBI is asymptomatic, it represents a potential reservoir for the transmission and progression of TB. The immune system plays a pivotal role in controlling the infection. In the majority of individuals, M.tb is controlled through the formation of granulomas, thereby preventing the proliferation of bacteria (4, 5).

The systemic immune-inflammation index (SIRI), calculated from the counts of neutrophils, monocytes, and lymphocytes in peripheral blood (6), has emerged as a promising tool for understanding the inflammatory processes underlying various diseases (7, 8). A clinical study involving 256 patients demonstrated that SIRI (>1.22) is a pivotal indicator for predicting the onset of coronary artery disease (9). A prospective study of 737 patients at Wuhan Jinyintan Hospital demonstrated that SIRI is a straightforward and effective inflammatory marker for predicting tuberculosis-related obstructive lung disease (10). A meta-analysis demonstrated that elevated SIRI was significantly associated with unfavorable prognosis in individuals diagnosed with colorectal cancer (hazard ratio [HR] = 2.65, 95% CI: 1.42-4.93). This suggests that SIRI may serve as an independent prognostic marker (11). Qian Zhou and colleagues observed that pretreatment SIRI may serve as a promising universal prognostic indicator in cancer (12). Tian Ren and colleagues demonstrated a statistically significant correlation between SIRI and all-cause mortality (HR = 2.18) as well as cardiovascular mortality (HR = 2.32) in patients with hyperuricemia (13).

SIRI is a newly developed composite measure that evaluates the overall level of inflammation in the body and has demonstrated significant promise as a prognostic indicator across various disease contexts. In the U.S. population, there was no known correlation between all-cause mortality and SIRI and LTBI. The present study, therefore, aimed to investigate the association between the SIRI and the risk of developing LTBI in U.S. adults through a population-based survey and to clarify the relationship between SIRI and all-cause mortality in patients with LTBI.

## 2 Methods

### 2.1 Study design and population

**Figure 1:**
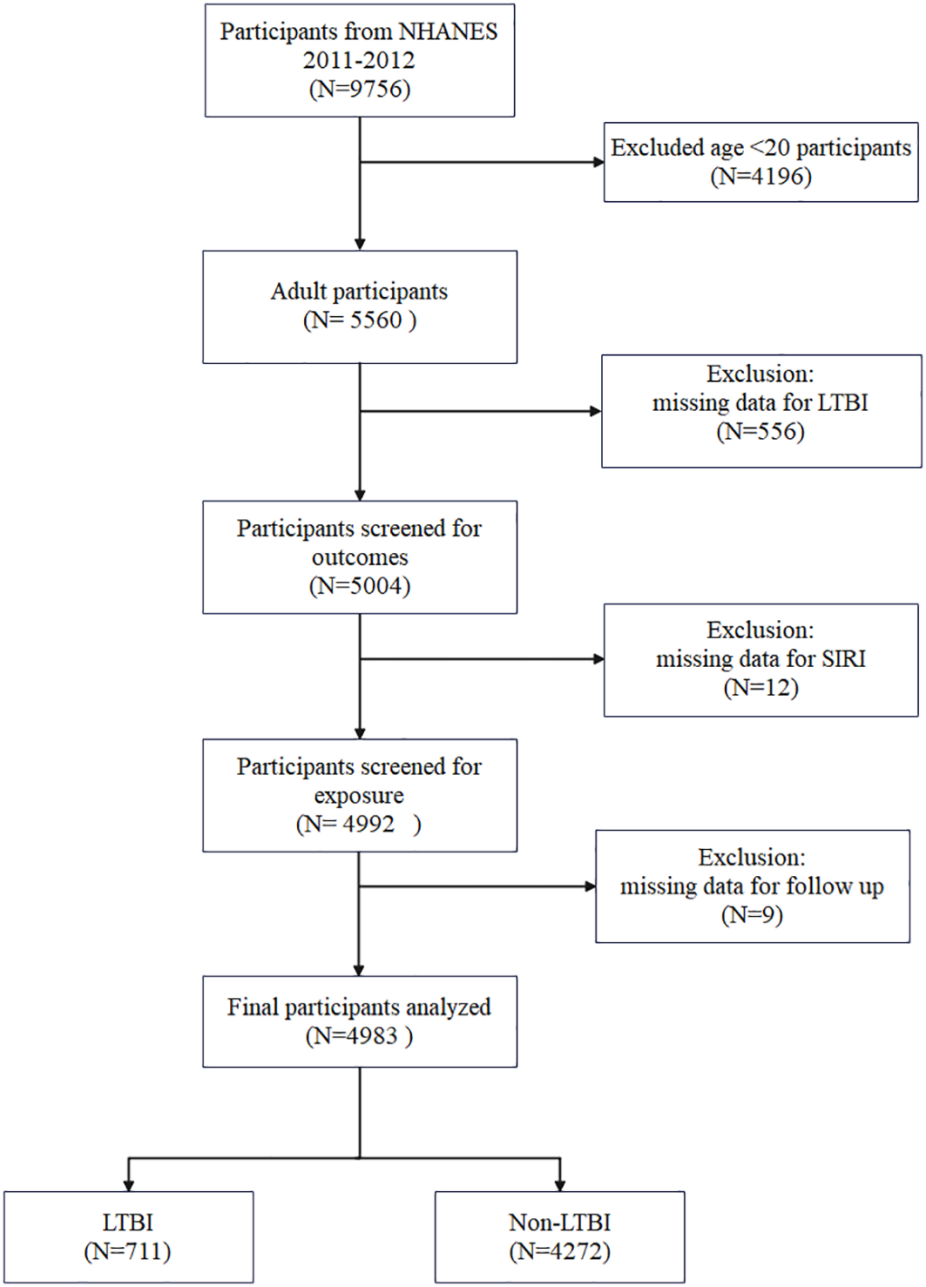
Flowchart of study.

The data for this study were obtained from the NHANES, an ongoing cross-sectional, multistage, stratified survey designed to assess the nutritional and health status of a nationally representative US civilian population. The database provided the data for this study, which was derived from interviews, health examinations, and laboratory measurements. The data pertaining to TB were recorded during the 2011–2012 period. Accordingly, this study solely employed publicly accessible data from NHANES 2011-2012. The present study included 9,756 participants during the period 2011–2012. Due to the limited availability of pertinent covariates, the study excluded individuals below the age of 20(N = 4196). Individuals lacking data on QuantiFERON^®^-TB Gold-In-Tube (QFT-GIT), TST, SIRI, and mortality were excluded. Finally, 4983 participants were enrolled, including 711 with LTBI and 4272 with Non-LTBI (**Figure 1**).

### 2.2 Definition of SIRI

SIRI is calculated from the counts of neutrophils, monocytes, and lymphocytes in peripheral blood. Peripheral blood samples of the NHANES participants were analyzed at the Mobile Examination Centers (MEC) using a Beckman Coulter HMX Hematology Analyzer. Lymphocyte, neutrophil, and monocyte counts were measured via complete blood count, and were presented as × se^3^ cells/uL. The calculation is as follows: SIRI= (segmented neutrophil count×monocyte count)/lymphocyte count (10). Therefore, the value of SIRI was expressed as ×spr cells/sed In this study, SIRI was employed as a continuous exposure variable, with its tertiles utilized as categorical variables. Participants were divided into three groups according to their SIRI value: T1 (SIRI < 0.73×10³ cells/0³), T2 (0.73×10³ cells/10 ≤ SIRI < 1.2×10³ cells/uL) and T3 (SIRI ≥ 1.2×10³ cells/³0).

### 2.3 Definition of LTBI

The diagnosis of LTBI is primarily based on the results of the tuberculin skin test (TST) and the interferon-gamma release assay (IGRA), also known as the QuantiFERON-TB Gold In-Tube (GFT-GIT) test. In this study, a positive result on the GFT-GIT or TST was taken to indicate the presence of LTBI (14–16). The positive results of TST were defined as induration size ≥ 10 mm. The following criteria must be met for the QuantiFERON-TB Gold assay to be interpreted as reactive: Nil value must be ≤ 8.0 IU gamma interferon (IF)/mL; TB antigen value minus Nil value must be ≥ 0.35 IU gamma interferon (IF)/mL.

### 2.4 Covariates

When referring to previous research, we included a number of factors as covariates. Gender, age, race, education levels, marital status, poverty-income ratio (PIR), BMI, smoke, alcohol use, hypertension, diabetes, hyperlipidaemia, and coronary heart disease (CHD) were all obtained from NHANES data. Gender, race, education levels, marital status, smoke, alcohol use, hypertension, diabetes, hyperlipidaemia, and CHD were all categorical variables, while age, PIR, and BMI were continuous variables. To facilitate comparison and analysis, the age variable was also divided into two groups: <60 years and ≥60 years. Smoke was identified as having smoked at least 100 cigarettes in a lifetime. Alcohol use was defined as at least 12 alcoholic beverages in 1 year. Participants were diagnosed with hypertension if they fulfilled any of the following conditions: 1) systolic blood pressure (SBP) ≥130 mmHg or diastolic blood pressure (DBP) ≥80 mmHg; 2) self-reported history of hypertension; 3) the use of antihypertensive medications (17). Participants were diagnosed with diabetes if they met any of the following criteria:1) self-reported diabetes; 2) use of insulin or other diabetes medications; 3) based on fasting plasma glucose≥ 7.0mmol/l; 4) glycosylated hemoglobin A1c (HbA1c) > 6.5% (18). “Having been told by a doctor or other health professional that you have a high blood cholesterol level” or “taking prescription drugs to lower cholesterol” is considered to have hyperlipidemia. The diagnosis of CHD was based on the affirmative response on MCQ 160c: ‘‘You’ve been diagnosed with coronary heart disease.’’

### 2.5 Assessment of all-cause mortality and follow-up

Mortality follow-up was performed for all participants aged ≥20 years, with the observation period extending through December 31, 2019. The study calculated follow-up duration in months, spanning from the initial mobile examination center assessment to either the participant’s date of death or the study’s terminal point. All-cause mortality was comprehensively evaluated, encompassing deaths attributed to any underlying etiology. The National Death index (NDI) linkage is an essential element in studies like those using NHANES data, especially when investigating long-term outcomes like mortality.

### 2.6 Statistical analysis

Continuous variables are presented as mean ± standard error(SE). Categorical variables are presented as number (percentage). Logistic regression model was employed to examine the association between SIRI and LTBI, with results presented as odds ratio (OR) and 95% confidence interval (CI). Kaplan-Meier curves were constructed for the purpose of evaluating survival over time. Multivariate Cox proportional hazard model was used to assess the association between SIRI and all-cause mortality, reporting hazard ratios (HR) with 95% confidence intervals (CI). To minimize potential bias and systematically assess the association between SIRI and all-cause mortality, three incremental statistical models were developed. The model 1 was unadjusted. Model 2 was adjusted to consider the impacts of gender and age. Model 3 was further additionally adjusted for race, education levels, marital status, PIR, BMI, smoking, alcohol consumption, hypertension, diabetes, hyperlipidaemia, and CHD. Furthermore, nonlinear relationships between SIRI and all-cause mortality were explored using restricted cubic spline (RCS) regression analysis. The present study did not employ sampling weights or the complex probability sample design included in the NHANES. Nevertheless, in order to guarantee the dependability of the findings, the results of the weighted analysis have been included in the **Supplementary Document**. All statistical analyses were performed with R software (version 4.2.2) and EmpowerStats (version 4.2). A statistically significant result was determined as a two-sided p-value<0.05. All statistical methods and results have been subjected to rigorous scrutiny to ensure accuracy and clarity.

## 3 Results

### 3.1 Sample characteristics

The study included a total of 4,983 participants, with an average age of 48.70 ± 17.69 years. Of these, 49.41% (n=2,462) were male. The mean follow-up period was 92.35 ± 16.82 months, and there were 525 deaths, representing a mortality rate of 10.54%. The participants were classified into three groups (T1, T2, and T3) based on their SIRI tertiles. Significant differences were observed across SIRI tertiles with regard to demographic and clinical characteristics. The T3 group exhibited higher proportions of males (55.36%), older age (52.16 ± 18.81 years), higher BMI (29.31 ± 7.27 kg/m²), and a greater prevalence of smoking (50.86%), hypertension (49.28%), diabetes (16.98%), and CHD (5.76%) compared to the other groups (all *P* value < 0.001). The baseline characteristics of the participants are detailed in **Table 1** and **Supplementary Table 1**. The weighted baseline characteristics of the study population are presented in **Supplementary Table 2**.

**Table 1:** Characteristics of the study population with various SIRI tertiles.

Characters	Total	SIRI Tertile			*P*-value
	(N=4983)	T1 group (N=1658)	T2 group (N=1647)	T3 group (N=1678)	
Gender, n(%)					<0.001
Male	2462 (49.41)	724 (43.67)	809 (49.12)	929 (55.36)	
Female	2521 (50.59)	934 (56.33)	838 (50.88)	749 (44.64)	
Age, years	48.70 ± 17.69	46.38 ± 16.64	47.50 ± 17.00	52.16 ± 18.81	<0.001
Age level					<0.001
<60	1567 (31.4)	423 (25.5)	474 (28.8)	670 (39.9)	
≥60	3416 (68.6)	1235 (74.5)	1173 (71.2)	1008 (60.1)	
Race, n(%)					<0.001
Mexican American	494 (9.91)	163 (9.83)	178 (10.81)	153 (9.12)	
Non-Hispanic White	1864 (37.41)	387 (23.34)	663 (40.26)	814 (48.51)	
Non-Hispanic Black	1279 (25.67)	636 (38.36)	354 (21.49)	289 (17.22)	
Other Race	1346 (27.01)	472 (28.47)	452 (27.44)	422 (25.15)	
Education levels, n(%)					0.003
Less than high school level	1150 (23.09)	48 (21.00)	386 (23.44)	416 (24.81)	
High school or equivalent	1048 (21.04)	349 (21.06)	317 (19.25)	382 (22.78)	
Greater than high school level	2783 (55.87)	960 (57.94)	944 (57.32)	879 (52.42)	
Marital status, n(%)					<0.001
Married/Living with partner	2819 (56.60)	898 (54.16)	987 (59.93)	934 (55.73)	
Widowed/Divorced/Separated/	1100 (22.08)	353 (21.29)	335 (20.34)	412 (24.58)	
Never married	1062 (21.32)	407 (24.55)	325 (19.73)	330 (19.69)	
PIR	2.44 ± 1.67	2.49 ± 1.66	2.52 ± 1.71	2.30 ± 1.62	<0.001
BMI, kg/m2	28.83 ± 6.89	28.37 ± 6.78	28.81 ± 6.58	29.31 ± 7.27	<0.001
Smoke, n(%)					<0.001
No	2840 (57.04)	1068 (64.49)	948 (57.59)	824 (49.14)	
Yes	2139 (42.96)	588 (35.51)	698 (42.41)	853 (50.86)	
Alcohol use, n(%)					<0.001
No	1183 (26.56)	438 (30.29)	367 (24.76)	378 (24.77)	
Yes	3271 (73.44)	1008 (69.71)	1115 (75.24)	1148 (75.23)	
Hypertension, n(%)					<0.001
No	2984 (59.88)	1081 (65.20)	1052 (63.87)	851 (50.72)	
Yes	1999 (40.12)	577 (34.80)	595 (36.13)	827 (49.28)	
Diabetes, n(%)					<0.001
No	4338 (87.06)	1470 (88.66)	1475 (89.56)	1393 (83.02)	
Yes	645 (12.94)	188 (11.34)	172 (10.44)	285 (16.98)	
Hyperlipidaemia, n(%)					0.726
No	3090 (62.34)	1014 (61.60)	1025 (62.46)	1051 (62.93)	
Yes	1867 (37.66)	632 (38.40)	616 (37.54)	619 (37.07)	
CHD, n(%)					<0.001
No	4796 (96.54)	1628 (98.19)	1598 (97.20)	1570 (94.24)	
Yes	172 (3.46)	30 (1.81)	46 (2.80)	96 (5.76)	
SIRI (10^3^cells/uL)	1.13 ± 0.83	0.50 ± 0.14	0.94 ± 0.13	1.94 ± 0.95	<0.001
LTBI, n(%)					0.003
No	4272 (85.73)	1393 (84.02)	1402 (85.12)	1477 (88.02)	
Yes	711 (14.27)	265 (15.98)	245 (14.88)	201 (11.98)	
Neutrophils (10^3^cells/uL)	4.12 ± 1.66	2.86 ± 0.93	4.00 ± 1.07	5.49 ± 1.66	<0.001
Neutrophils percent (%)	58.30 ± 9.88	50.25 ± 8.66	58.71 ± 6.78	65.84 ± 7.04	<0.001
Monocyte (10^3^ cells/uL)	0.51 ± 0.24	0.39 ± 0.29	0.49 ± 0.13	0.64 ± 0.19	<0.001
Lymphocyte(10^3^ cells/uL)	2.07 ± 1.07	2.24 ± 1.59	2.08 ± 0.66	1.90 ± 0.67	<0.001
Platelet (10^3^ cells/uL)	236.14 ± 60.56	227.94 ± 54.51	236.81 ± 59.32	243.58 ± 66.21	<0.001
All-cause mortality, n(%)					<0.001
No	4458 (89.46)	1552 (93.61)	1517 (92.11)	1389 (82.78)	
Yes	525 (10.54)	106 (6.39)	130 (7.89)	289 (17.22)	
Follow-up time (months)	92.35 ± 16.82	93.91 ± 13.10	93.52 ± 15.01	89.65 ± 20.94	<0.001

BMI, body mass index; PIR, family poverty to income ratio; CHD, Coronary heart disease; Continuous variables are presented as mean (standard error). Categorical variables are presented as n (%). Univariate logistic regression models were used for continuous and categorical variables.

### 3.2 Associations between SIRI and LTBI

As shown in **Table 1**, those with higher SIRI values had a lower prevalence of LTBI (T1 group:15.98% vs. T2 group:14.88% vs. T3 group: 11.98%). Logistic regression analysis showed that SIRI was significantly inversely associated with the occurrence of LTBI. In Model 1 (unadjusted), the risk of developing LTBI reduced by 26% for every one-unit increase in the SIRI (OR=0.74, 95% CI: 0.65–0.84). After adjusting for sex and age (Model 2), a one-unit increase in SIRI was associated with a 34% reduction in the risk of developing LTBI (OR=0.66, 95% CI: 0.58–0.75). After full adjustment (Model 3), maintains significance (OR=0.76, 95% CI: 0.66–0.89). The T3 groups with higher SIRI values had a lower risk of LTBI than did the T1 group (Model 3: OR=0.72, 95% CI: 0.56-0.93). The trend across tertiles is statistically significant (*P* < 0.05) (**Table 2**).Furthermore, in order to provide additional verification of the stability of the results, weighted multivariate logistic regression was employed for the purpose of evaluating the association between SIRI and LTBI prevalence. The findings showed that there was still a negative association between SIRI and LTBI (OR=0.80, 95% CI: 0.67- 0.963). As detailed in **Supplementary Table 3**.

**Table 2:** Associations between SIRI and LTBI.

Variable	Model1		Model2		Model3	
	OR(95%CI)	*P*-value	OR(95%CI)	*P*-value	OR(95%CI)	*P*-value
SIRI	0.74 (0.65, 0.84)	<0.001	0.66 (0.58, 0.75)	<0.001	0.76 (0.66, 0.89)	<0.001
SIRI tertile						
T1 (<0.73)	Ref.		Ref.		Ref.	
T2 (0.73-1.2)	0.92 (0.76, 1.11)	0.378	0.87 (0.72, 1.06)	0.168	0.95 (0.76, 1.20)	0.693
T3 (>1.2)	0.72 (0.59, 0.87)	0.001	0.59 (0.48, 0.73)	<0.001	0.72 (0.56, 0.93)	0.011
*P* for trend		<0.001		<0.001		0.012

Ref, Reference; OR, Odds Ratio; CI, Confidence Interval; Model1: unadjusted; Model2: adjusted for gender and age; Model 3: adjusted for gender, age, race, education levels, marital status, PIR, BMI, smoke, alcohol use, hypertension, diabetes, hyperlipidaemia, and CHD.

An analysis of the correlation between immune cell counts from routine blood tests and LTBI is included in the **Supplementary Material**. Using univariate analysis, we found that monocytes and neutrophils showed a significant decrease in the LTBI population compared to the non-LTBI population (*P* < 0.05). However, other parameters, including lymphocyte count, eosinophils, and basophils, did not demonstrate significant differences (**Supplementary Table 4**). A multivariate logistic regression analysis was conducted, revealing a significant association between decreased monocyte counts and the occurrence of LTBI (**Supplementary Table 5**).

### 3.3 Associations between SIRI and all-cause mortality in patients with LTBI

**Figure 2:**
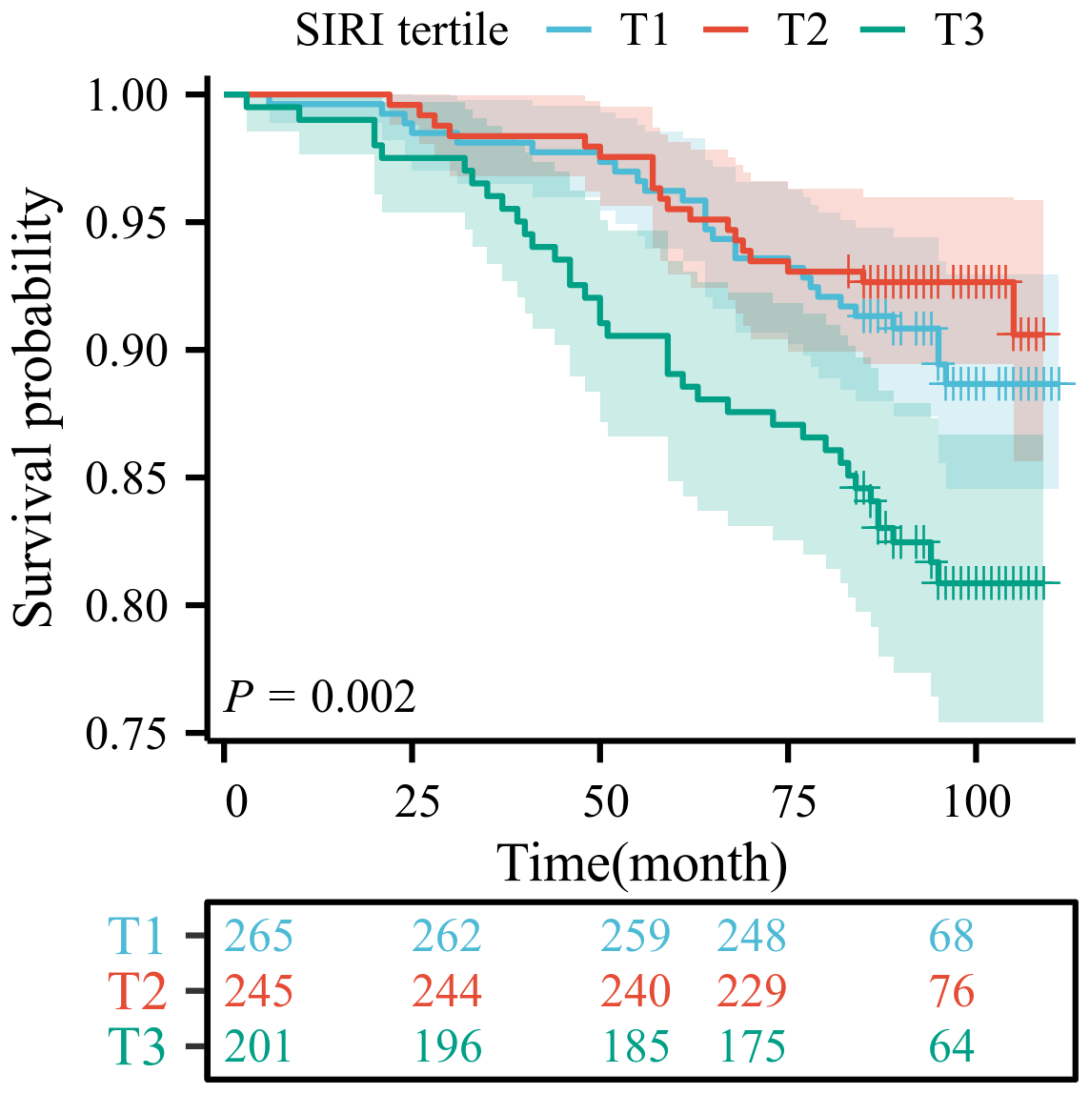
Kaplan-Meier survival curve for the population with LTBI.

Among patients with LTBI, each unit increase in SIRI was associated with a 48% higher risk of mortality (Model 3: HR=1.48, 95% CI:1.01-2.15). The T3 group demonstrated a statistically significant correlation with mortality outcomes only in Model 1 (HR=1.90, 95% CI: 1.15-3.11), as detailed in **Table 3**. To ensure the reliability of the results, we performed a weighted multivariate Cox regression analysis, which showed consistent findings. Higher SIRI levels was associated with an increased risk of death, as detailed in **Supplementary Tables 6**-**8**. The Kaplan-Meier survival curve analysis revealed a significant difference in survival probability among the tertiles of SIRI (T1, T2, T3) (*P* = 0.002). The survival probability of the high SIRI group (T3) was significantly lower than that of the low SIRI group (T1). This finding indicates that SIRI is a significant predictor of prognosis in patients with LTBI (**Figure 2**).

**Table 3:** The relationships between SIRI and all-cause mortality in LTBI.

Variable	Model1		Model2		Model3	
	HR (95%CI)	*P*-value	HR (95%CI)	*P*-value	HR (95%CI)	*P*-value
SIRI	1.50 (1.18,1.91)	0.001	1.37 (1.03,1.83)	0.029	1.48 (1.01,2.15)	0.043
SIRI tertile						
T1 (<0.73)	Ref.		Ref.		Ref.	
T2 (0.73-1.2)	0.75 (0.42,1.35)	0.342	0.71 (0.39,1.27)	0.248	0.63 (0.31,1.27)	0.199
T3 (>1.2)	1.90 (1.15,3.11)	0.012	1.22 (0.73,2.03)	0.448	1.30 (0.71,2.39)	0.400

Ref, Reference; HR, Hazard Ratio; CI, Confidence Interval; Model1: unadjusted; Model2: adjusted for gender and age; Model3: adjusted for gender, age, race, education levels, marital status, PIR, BMI, smoke, alcohol use, hypertension, diabetes, hyperlipidaemia, and CHD.

The results of the association between LTBI and all-cause mortality, as well as the analysis of the association between the SIRI and all-cause mortality in both the overall population and the non-LTBI population, are presented in the **Supplementary Material**. The association between LTBI and all-cause mortality did not demonstrate statistical significance in the multivariable Cox proportional hazards model (*P* = 0.662), as illustrated in **Supplementary Table 9**. SIRI demonstrated a significant association with all-cause mortality among the entire cohort, with a considerably elevated risk of death in the high SIRI group (T3) (P < 0.001), a finding that persisted after adjusting for confounding factors (HR = 1.46, 95% CI: 1.12-1.91). In participants without LTBI, SIRI demonstrated a significant association with all-cause mortality, with the highest risk observed in the high SIRI group (T3) (*P* < 0.001), and the adjusted risk remained significant (HR = 1.81, 95% CI: 1.34-2.44). Please refer to the data presented in **Supplementary Table 10**. The Kaplan-Meier survival curve analysis across all populations demonstrated that elevated SIRI levels (specifically, the T3 group) were significantly associated with a reduced probability of survival (**Supplementary Figure 1**).

Additionally, the analysis of the association between baseline immune cell parameters and all-cause mortality in patients with LTBI was included. Univariate analysis demonstrated that lymphocyte count, lymphocyte percentage, monocyte percentage, and basophil count exhibited statistically significant associations with all-cause mortality in patients with LTBI (*P* < 0.05; **Supplementary Table 11**). Multivariate Cox regression analysis further demonstrated that lymphocyte count exhibited a significant association with mortality in the unadjusted model (HR = 0.47, *P* < 0.001), although this correlation diminished after adjusting for confounding factors (*P* > 0.05). Conversely, no significant associations were observed between other immune cell parameters and all-cause mortality in the multivariate model (**Supplementary Table 12**).

### 3.4 Restricted cubic splines analysis of the association between SIRI and all-cause mortality in patients with LTBI

**Figure 3:**
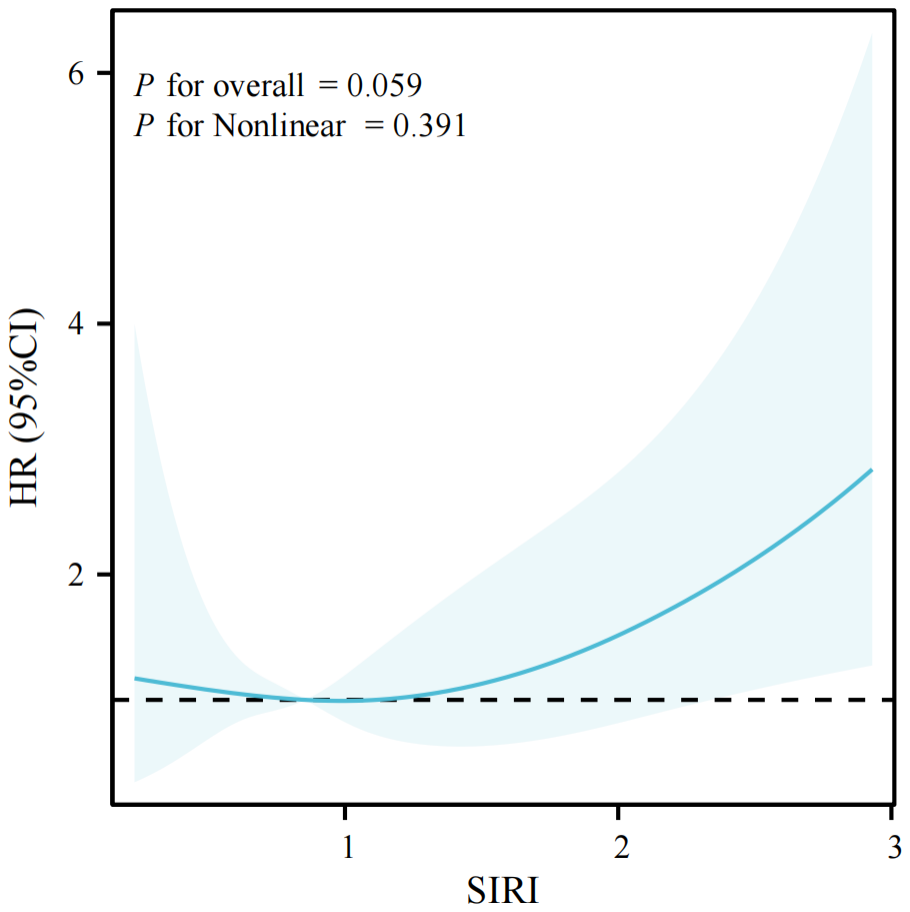
The association of SIRI with all-cause among LTBI visualized by restricted cubic spline.

In this study, we conducted a series of restricted cubic spline analyses to investigate the association between the SIRI and all-cause mortality in patients with LTBI. The findings demonstrated a significant linear correlation between SIRI and all-cause mortality (*P* for nonlinear > 0.05) after adjusting for confounding variables (see **Figure 3**). The association between SIRI and all-cause mortality among all participants included in the study is illustrated by a restricted cubic spline in **Supplementary Figure 2**. A further stratified analysis was conducted by age and sex, revealing consistent linear associations across younger (< 60 years) and older (l 60 years) participants, as well as among male and female subjects (*P* for adjusted non-linearity > 0.05). Further visualizations can be found in **Supplementary Figures 3**, **4**.

### 3.5 The predictive ability of SIRI for all-cause mortality in patients with LTBI

**Figure 4:**
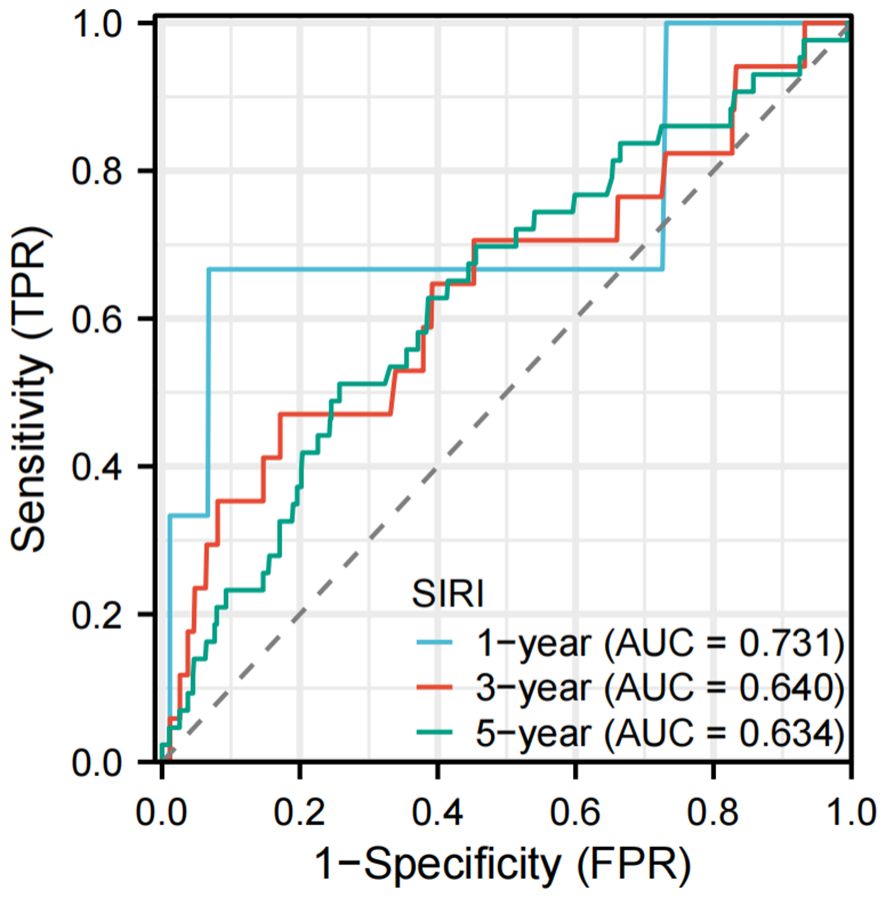
Time-dependent ROC curves of the SIRI for predicting all-cause mortality in participants with LTBI. AUC, area under the curve; ROC, receiver operating characteristic.

The data were analyzed using the “timeROC” package, and the results were visualized with the “ggplot2” package. As shown in **Figure 4**, the AUC values are 0.731 (1-year), 0.640 (3-year), and 0.634 (5-year) in the LTBI population. **Supplementary Figure 5** presents the time-dependent ROC curves of the SIRI in predicting all-cause mortality among the entire cohort of participants.

### 3.6 Subgroup analysis

**Figure 5:**
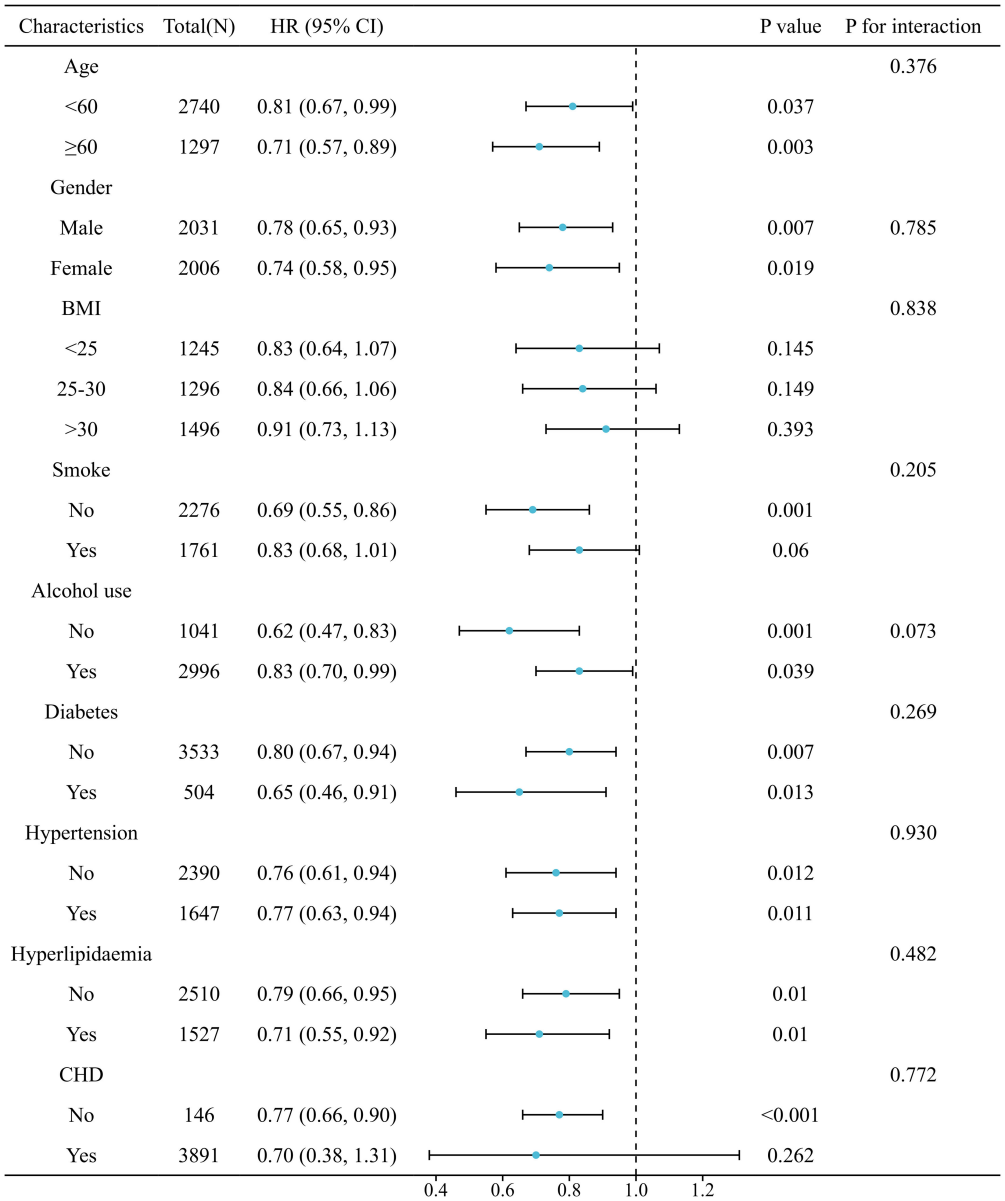
Stratified analysis of relationship between SIRI and risk of LTBI. (Adjusted for gender, age, race, education levels, marital status, PIR, BMI, smoke, alcohol use, hypertension, diabetes, hyperlipidaemia, and CHD).

**Figure 6:**
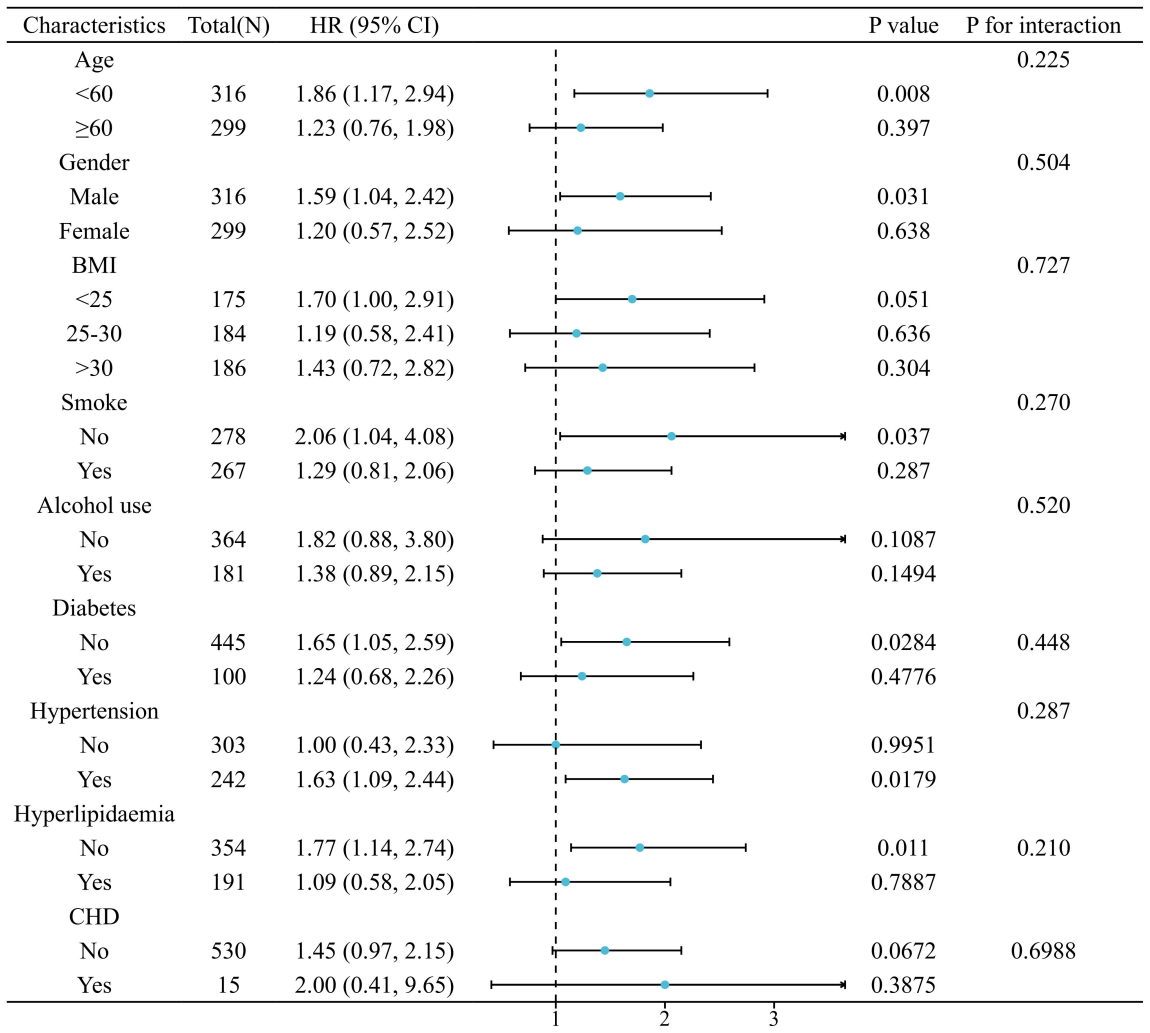
Stratified analysis of relationship between SIRI and risk of all-cause mortality in patients with LTBI. (Adjusted for gender, age, race, education levels, marital status, PIR, BMI, smoke, alcohol use, hypertension, diabetes, hyperlipidaemia, and CHD).

The relationship between SIRI and LTBI was consistent across all analyzed subgroups (*P* for interaction > 0.05), as illustrated in **Figure 5**. The correlation between SIRI levels and all-cause mortality in patients with LTBI persisted across all analyzed subgroups (*P* for interaction > 0.05), as demonstrated in **Figure 6**. However, a range of demographic and lifestyle factors, including age, gender, BMI, alcohol use, and diabetes status, varied significantly across all populations (see **Supplementary Figure 6**).

## 4 Discussion

This study, based on the NHANES 2011-2012 database, provides the first comprehensive insight into the complex associations of the SIRI with LTBI and all-cause mortality. During the mean follow-up period of 92.35 months, a significant inverse association was identified between SIRI and LTBI (adjusted OR=0.76, 95% CI: 0.66-0.89). The observed association was consistent across gender, age group, BMI group, smoking status, alcohol consumption, and the presence of diabetes, hypertension, hyperlipidaemia, and CHD. Conversely, a significant positive association was observed between SIRI and all-cause mortality. Among patients with LTBI, each unit increase in SIRI was associated with a 48% higher risk of mortality (adjusted HR=1.48, 95% CI:1.01-2.15). The linear dose-response relationship was confirmed by RCS. The time-dependent ROC curve demonstrated that SIRI exhibited moderate predictive capacity for all-cause mortality. In the LTBI population, SIRI demonstrated AUCs of 0.731, 0.640, and 0.634 for predicting all-cause mortality at 1, 3, and 5 years, respectively.

The present study found that the mean age of participants in the highest SIRI tertile (T3) was significantly greater than that of those in the lower tertiles (T1 and T2). This finding is consistent with the report by Accardi G. et al. (19), which indicated that elevated SIRI levels correlate with advancing age (19). This phenomenon may reflect age-related immune dysregulation and the progressive accumulation of chronic low-grade inflammation, commonly referred to as “inflammaging” (20). However, when grouping participants by age at 60, the proportion of T3 was higher among individuals younger than 60, while the proportion of T1/T2 was greater among those aged 60 and older. This seemingly paradoxical phenomenon may reflect the varied inflammatory regulatory mechanisms present in different age groups. In the context of global aging, the number of individuals aged 60 and older is increasing rapidly, leading to a sharp rise in the prevalence of chronic diseases among the elderly and a growing number of individuals facing multiple coexisting diseases (21). Therefore, it is possible that the elderly population received anti-inflammatory or immunomodulatory therapies for multiple comorbidities, which may have contributed to the reduced proportion of T3 in the SIRI group. Additionally, the sample of the elderly population may have been underestimated due to survival bias; that is, individuals with high SIRI may have died from complications (22) and were not included in the cross-sectional study. Chronic low-grade inflammation is associated with increased rates of various age-related diseases and all-cause mortality (23–25). According to a study based on the NHANES, elevated systemic immune inflammation index (SII) and SIRI were significantly associated with all-cause mortality risk in the general population aged 60 and older. This suggests that as SII or SIRI levels increase, the risk of all-cause mortality in the elderly correspondingly rises (26). Additionally, a meta-analysis showed that high SIRI was significantly associated with both short-term and long-term mortality in stroke patients (27).

In recent years, the potential value of routine blood indicators for disease prediction has gradually attracted attention alongside the development of precision medicine. A study published in *Nature* in 2025 demonstrated that long-term stable set points of individual routine blood indicators can serve as biomarkers to predict the risk of various diseases, including cardiovascular disease, diabetes, and atrial fibrillation, thereby providing a new approach for early screening and personalized health management (28). An increased SIRI level indicates an intensified inflammatory state, which may result from an augmented neutrophil and monocyte count and/or a decreased lymphocyte count. LTBI is characterized by a latent immune response to M.tb, in which T-cell-mediated immunity plays a core role in controlling bacterial replication (29). Intriguingly, in the present study, we observed that lower SIRI levels were associated with the prevalence of LTBI in U.S. adults, even after adjusting for confounding variables. Similar to our study, previous research has found an inverse association between the neutrophil-to-lymphocyte ratio and the likelihood of LTBI (30). The underlying mechanisms linking SIRI to LTBI remain unclear. The association between the two may be due to the fact that the occurrence of M.tb infection is related to an individual’s low-grade inflammatory state. We speculate that this is primarily because the low-grade inflammatory state may weaken the immune function, making it easier for M.tb to invade and multiply. Evidence suggests that low-grade inflammation is associated with chronic diseases and metabolic disorders (31, 32). For example, patients with diabetes mellitus often experience chronic low-grade inflammation, which affects immune cell function, particularly the reduced activity of alveolar macrophages, thereby increasing the risk of infection (33).

The immune system is fundamental to human health, as most diseases and health conditions are closely linked to immune status. Routine blood parameters, including lymphocyte, monocyte, neutrophil, eosinophil, and basophil counts, are commonly used to assess immune cell status. This study further investigated the association between peripheral blood immune cell counts and the occurrence of LTBI (see **Supplementary Tables 4**, **5**). Our analysis revealed significantly lower monocyte and neutrophil counts in individuals with LTBI compared to non-LTBI controls (*P* < 0.05), with monocyte depletion showing a particularly strong association with LTBI development. This finding aligns with the critical role of monocytes in the immunoregulation of TB. Monocytes serve as precursors to macrophages, the primary phagocytic cells responsible for engulfing and restricting the growth of M.tb. Additionally, monocytes modulate inflammatory responses that influence disease progression. Reduced monocyte counts may compromise the host’s capacity for bacterial clearance. Notably, certain medications, such as glyburide, may exacerbate this vulnerability by suppressing monocyte activity (34). Recent transcriptomic analyses of monocyte-related genes revealed increased monocyte abundance in patients with active TB, while individuals with LTBI exhibited 165 differentially expressed monocyte-associated genes. These genes were enriched in pathways related to the inflammatory response and immune regulation, potentially influencing the establishment of latent infection through metabolic reprogramming or modulation of complement system activity (35). M.tb infection significantly alters the monocyte-to-lymphocyte ratio (MLR) in peripheral blood, with extreme MLR (<9% or >25%) serving as critical predictors of active TB (36).

However, since this part of the study is based on cross-sectional data, we cannot assess the causal relationship between low SIRI levels and M.tb infection. The association between the two may also be related to dynamic changes in the immune and inflammatory states associated with LTBI. Previous studies have indicated that LTBI is typically accompanied by a sustained, long-term low-grade inflammatory state, rather than a notable systemic inflammatory response (37). Moreover, long-term immune activation in individuals with LTBI might increase the risk of hypertension and atherosclerotic cardiovascular diseases (CVD) (38, 39). High SIRI values might be more related to other acute or chronic inflammatory diseases (such as CVD, chronic kidney disease, acute gout attacks, etc.) rather than LTBI (40–42). Additionally, SIRI values might not only be associated with the risk of LTBI but also predict the risk of LTBI progressing to active TB (43). Xiaoshan He et al. research found that the SIRI is an important inflammatory indicator for predicting cavitary lesions in pulmonary TB. A SIRI value higher than 2.095 is significantly associated with cavitary pulmonary tuberculosis, and the combination of CRP and PLR can significantly enhance the diagnostic sensitivity for cavitary pulmonary tuberculosis (44). This suggests that SIRI, as an inflammatory marker, may play an important role in immune monitoring and in predicting disease progression in LTBI. In the future, prospective cohort studies could be conducted to monitor changes in SIRI at different stages of TB (incubation, progressive, and active) and to verify its value as a predictor.

In the present study, no significant associations were observed between monocyte count, lymphocyte count, and neutrophil count and all-cause mortality in patients with LTBI (see **Supplementary Tables 11**, **12**). However, a composite measure of these three cell types, the SIRI, demonstrated a significant association with all-cause mortality. This finding suggests that the relationship between cell counts alone and mortality may be obscured by other factors, and that SIRI, as a composite indicator, can more effectively reflect the overall health status of patients.

SIRI has emerged as a novel and cost-effective serum biomarker for predicting all-cause mortality. The observed positive association between elevated SIRI levels and increased mortality aligns with findings from previous research. Numerous studies have demonstrated that higher SIRI levels are linked to an increased risk of all-cause mortality across various conditions, including asthma (45), chronic kidney disease (46), hypertension (47), and heart failure (48). Our findings further support this association, showing that elevated SIRI levels are significantly correlated with increased all-cause mortality risk in patients with LTBI. As an integrative marker of inflammation and immune function, SIRI reflects the dynamic interplay between neutrophils, monocytes, and lymphocytes. Elevated SIRI levels indicate a heightened inflammatory state and may serve as a potential predictor of cardiovascular disease (38–41). In patients with LTBI and high SIRI levels, persistent inflammation could exacerbate comorbidities such as cardiovascular disease and diabetes, ultimately increasing mortality risk (49, 50). Research has indicated that diminished monocyte counts and elevated lymphocyte-to-monocyte ratios are significantly associated with unfavorable outcomes in TB (51). Estevez O. et al. conducted a retrospective analysis of 52 patients with tuberculosis and found that a lower monocyte count and a higher lymphocyte-to-monocyte ratio were significantly associated with an increased risk of death within 3 months (52). They also found that longitudinal changes in monocyte counts serve as prognostic indicators.

Despite the absence of direct data from patients with active TB in the present study, a comprehensive review of the existing literature revealed the significant potential value of inflammatory markers such as the SIRI, MLR, neutrophil-to-lymphocyte ratio (NLR), and CD64 expression in distinguishing between different types of TB and its active state. For instance, SIRI levels were found to be significantly higher in patients with cavitary pulmonary TB than in those with non-cavitary pulmonary TB, suggesting that SIRI may serve as a potential biomarker to distinguish between these two types of pulmonary TB (44). Chai et al. demonstrated that SIRI levels were elevated in patients with pulmonary TB compared to those with non-TB pulmonary infections (53). Furthermore, MLR has been shown to be significantly higher in patients with active TB than in those with LTBI and healthy donors. The MLR and CD64 expression have been identified as effective biomarkers that can help distinguish active TB from other states (54). Single-cell RNA sequencing and flow cytometry have revealed an increased proportion of monocytes in patients with active TB (55). The study by Fritschi N et al. demonstrated that elevated MLR and NLR were significantly associated with M.tb infection compared with healthy controls (56). Furthermore, studies have demonstrated that SIRI can be utilized as a biomarker for expedited screening of multidrug-resistant TB when combined with the cardiometabolic index, yielding an AUC value of 0.910 in patients with new-onset TB (57). These findings underscore the significance of inflammatory markers such as SIRI in the diagnosis and prognostic assessment of TB.

This study has several significant advantages. Firstly, the nationally representative sample based on the NHANES database ensures the external validity of the research findings. Secondly, the research employs rigorous statistical methods, including multivariate adjustment, subgroup analysis, and restricted cubic spline analysis, thereby enhancing the reliability of the findings. Thirdly, this is the inaugural study to investigate the correlation between SIRI and LTBI, thereby offering a novel perspective for comprehending the function of inflammatory responses in tuberculosis infection. Furthermore, the relatively long follow-up period (with an average of 92.35 months) allows for the reliable assessment of the relationship between SIRI and long-term prognosis.

There are certain limitations to our study. Firstly, it is not possible to determine the causal relationship between SIRI and LTBI. Secondly, SIRI was derived from a single complete blood count rather than multiple measurements over time, which may have introduced bias. Thirdly, although multivariate adjustment was conducted, there may still be unidentified confounding factors. Fourthly, the study was unable to obtain comprehensive medication data from the participants, which may have influenced the observed levels of inflammatory markers. In addition, the use of Mtb sensitization (as measured by TST or QFT) as a proxy for LTBI is a limitation in TB research and clinical practice. In conclusion, due to the nature of the NHANES database, the study results are primarily applicable to the U.S. population. Further verification is required to evaluate the generalizability of the findings in other populations.

## 5 Conclusion

Our findings reveal an inverse correlation between SIRI and LTBI in the U.S. adult population, with higher SIRI levels associated with a lower risk of LTBI. However, in individuals with LTBI, a higher SIRI was associated with an elevated risk of all-cause mortality.

## Data Availability

The original contributions presented in the study are included in the article/**Supplementary Material**. Further inquiries can be directed to the corresponding author.
