# Supplementary material for: Association of systemic inflammation response index with latent tuberculosis infection and all-cause mortality: a cohort study from NHANES 2011-2012
Source: Front Immunol. 2025 Feb 19;16:1538132. doi: 10.3389/fimmu.2025.1538132 (PMC11880221; doi:10.3389/fimmu.2025.1538132)
Supplement: Supplementary file 1 [file DataSheet1.docx]

**Table Legends**

Supplementary Table 1. Baseline characteristics of patients were divided into survival and death groups.

Supplementary Table 2. Weighted baseline characterization.

Supplementary Table 3. Weighted multivariable logistic regression for association between SIRI and LTBI.

Supplementary Table 4. Univariate analysis of immune cell parameters in bblood tests related to LTBI.

Supplementary Table 5. Multivariable logistic regression for association between immune cell parameters and LTBI.

Supplementary Table 6. Weighted Multivariate Cox proportional hazard models for association between SIRI and all-cause mortality.

Supplementary Table 7. Weighted Multivariate Cox proportional hazard models for association between SIRI and all-cause mortality in LTBI patients.

Supplementary Table 8. Weighted multivariate Cox proportional hazard models for association between SIRI and all-cause mortality in non-LTBI patients.

Supplementary Table 9. Associations of SIRI with all-cause mortality in all populations and Non-LTBI populations.

Supplementary Table 10. Multivariate Cox proportional hazard models for association between LTBI and all-cause mortality.

Supplementary Table 11. Univariate analysis of baseline immune cell parameters and all-cause mortality in patients with LTBI.

Supplementary Table 12. Multivariate Cox proportional hazard models for association between immune cell parameters and all-cause mortality in patients with LTBI.

**Figure Legends**

Supplementary Figure 1. Kaplan-Meier survival curve for all-cause of all included participants.

Supplementary Figure 2. The association of SIRI with all-cause among all included participants visualized by restricted cubic spline.

Supplementary Figure 3. Restricted cubic spline analyses the association of SIRI with all-cause mortality in sex and age subgroups across all populations.

Supplementary Figure 4. Restricted cubic spline analyses the association of SIRI with all-cause mortality in sex and age subgroups with LTBI populations.

Supplementary Figure 5. Time-dependent ROC curves of the SIRI for predicting all-cause mortality among all included participants.

Supplementary Figure 6 . Stratified analysis of relationship between SIRI and risk of all-cause mortality in all patients.

**Supplementary Table 1. Baseline characteristics of patients were divided into survival and death groups.**

| **Characters** | **Total** | **Survival group** | **Death group** | ***P* value** |
| --- | --- | --- | --- | --- |
|  | **(N=4983)** | **（N=4458)** | **(N=525)** |  |
| **Gender, n(%)** |  |  |  | <0.001 |
| Male | 2462 (49.41%) | 2161 (48.47%) | 301 (57.33%) |  |
| Female | 2521 (50.59%) | 2297 (51.53%) | 224 (42.67%) |  |
| **Age, years** | 48.70 ± 17.69 | 46.34 ± 16.57 | 68.66 ± 13.90 | <0.001 |
| **Race, n(%)** |  |  |  | <0.001 |
| Mexican American | 494 (9.91%) | 467 (10.48%) | 27 (5.14%) |  |
| Non-Hispanic White | 1864 (37.41%) | 1586 (35.58%) | 278 (52.95%) |  |
| Non-Hispanic Black | 1279 (25.67%) | 1137 (25.50%) | 142 (27.05%) |  |
| Other Race | 1346 (27.01%) | 1268 (28.44%) | 78 (14.86%) |  |
| **Education levels, n(%)** |  |  |  | <0.001 |
| Less than high school level | 1150 (23.09%) | 953 (21.38%) | 197 (37.67%) |  |
| High school or equivalent | 1048 (21.04%) | 919 (20.61%) | 129 (24.67%) |  |
| Greater than high school level | 2783 (55.87%) | 2586 (58.01%) | 197 (37.67%) |  |
| **Marital status, n(%)** |  |  |  | <0.001 |
| Married/Living with partner | 2819 (56.60%) | 2561 (57.46%) | 258 (49.24%) |  |
| Widowed/Divorced/Separated/ | 1100 (22.08%) | 884 (19.83%) | 216 (41.22%) |  |
| Never married | 1062 (21.32%) | 1012 (22.71%) | 50 (9.54%) |  |
| **PIR** | 2.44 ± 1.67 | 2.48 ± 1.68 | 2.05 ± 1.45 | <0.001 |
| **BMI, kg/m^2^** | 28.83 ± 6.89 | 28.85 ± 6.89 | 28.66 ± 6.92 | <0.001 |
| **Smoke, n(%)** |  |  |  | <0.001 |
| No | 2840 (57.04%) | 2619 (58.80%) | 221 (42.10%) |  |
| Yes | 2139 (42.96%) | 1835 (41.20%) | 304 (57.90%) |  |
| **Alcohol use, n(%)** |  |  |  | 0.051 |
| No | 3271 (73.44%) | 1036 (26.11%) | 147 (30.25%) |  |
| Yes | 1183 (26.56%) | 2932 (73.89%) | 339 (69.75%) |  |
| **Hypertension, n(%)** |  |  |  | <0.001 |
| No | 2984 (59.88%) | 2819 (63.23%) | 165 (31.43%) |  |
| Yes | 1999 (40.12%) | 1639 (36.77%) | 360 (68.57%) |  |
| **Diabetes, n(%)** |  |  |  | <0.001 |
| No | 4338 (87.06%) | 3954 (88.69%) | 384 (73.14%) |  |
| Yes | 645 (12.94%) | 504 (11.31%) | 141 (26.86%) |  |
| **Hyperlipidaemia, n(%)** |  |  |  | 0.163 |
| No | 3090 (62.34%) | 2750 (62.01%) | 340 (65.13%) |  |
| Yes | 1867 (37.66%) | 1685 (37.99%) | 182 (34.87%) |  |
| **CHD, n(%)** |  |  |  | <0.001 |
| No | 172 (3.46%) | 96 (2.16%) | 76 (14.73%) |  |
| Yes | 4796 (96.54%) | 4356 (97.84%) | 440 (85.27%) |  |
| **SIRI** | 1.13 ± 0.83 | 1.07 ± 0.70 | 1.63 ± 1.42 | <0.001 |
| **SIRI tertile** |  |  |  | <0.001 |
| T1 | 1658 (33.27%) | 1552 (34.81%) | 106 (20.19%) |  |
| T2 | 1647 (33.05%) | 1517 (34.03%) | 130 (24.76%) |  |
| T3 | 1678 (33.67%) | 1389 (31.16%) | 289 (55.05%) |  |
| **LTBI, n(%)** |  |  |  | 0.286 |
| No | 4272 (85.73%) | 3830 (85.91%) | 442 (84.19%) |  |
| Yes | 711 (14.27%) | 628 (14.09%) | 83 (15.81%) |  |
| **Neutrophils (10^3^cells/uL)** | 4.12 ± 1.66 | 4.08 ± 1.64 | 4.44 ± 1.83 | <0.001 |
| **Neutrophils percent (%)** | 58.30 ± 9.88 | 57.92 ± 9.60 | 61.49 ± 11.54 | <0.001 |
| **Monocyte (10^3^ cells/uL)** | 0.51 ± 0.24 | 0.50 ± 0.19 | 0.58 ± 0.48 | <0.001 |
| **Lymphocyte(10^3^ cells/uL)** | 2.07 ± 1.07 | 2.08 ± 0.66 | 1.99 ± 2.69 | 0.063 |
| **Platelet (10^3^ cells/uL)** | 236.14 ± 60.56 | 238.10 ± 59.13 | 219.48 ± 69.46 | <0.001 |
| **Follow-up time (months)** | 92.35 ± 16.82 | 96.84 ± 6.62 | 54.20 ± 26.19 | <0.001 |

BMI= body mass index; CHD= Coronary heart disease; PIR= family poverty to income ratio; SIRI=systemic inflammation response index.Continuous variables are presented as mean±SE. Categorical variables are presented as n (%). Univariate logistic regression models were used for continuous and categorical variables.

**Supplementary Table 2. Weighted baseline characterization.**

| **Characteristic** | **Non-LTBI** | **LTBI** | ***P* value** |
| --- | --- | --- | --- |
|  |  |  |  |
| **Weighted N** | 188,257,219 | 16,919,815 |  |
| **Unweighted n** | 4,272 | 711 |  |
| **Gender** |  |  | 0.007 |
| Female | 2,210 (52.5%) | 311 (45.5%) |  |
| Male | 2,062 (47.5%) | 400 (54.5%) |  |
| **Age** | 46 (33, 59) | 52 (38, 62) | 0.002 |
| **Education levels** |  |  | <0.001 |
| Less than high school level | 893 (14.7%) | 257 (32.0%) |  |
| High school or equivalent | 901 (19.6%) | 147 (22.1%) |  |
| Greater than high school level | 2,476 (65.7%) | 307 (45.9%) |  |
| **Marital status** |  |  | 0.012 |
| Married/Living with partner | 2,371 (62.0%) | 448 (62.6%) |  |
| Widowed/Divorced/Separated | 921 (18.0%) | 179 (24.4%) |  |
| Never married | 978 (20.1%) | 84 (13.0%) |  |
| **Race** |  |  | <0.001 |
| Mexican American | 387 (6.8%) | 107 (18.6%) |  |
| Non-Hispanic Black | 1,109 (10.7%) | 170 (15.0%) |  |
| Non-Hispanic White | 1,777 (70.3%) | 87 (30.7%) |  |
| Other Race | 999 (12.1%) | 347 (35.7%) |  |
| **PIR** | 2.93 (1.34, 5.00) | 1.85 (0.95, 3.67) | <0.001 |
| **BMI** | 28 (24, 32) | 28 (24, 32) | 0.936 |
| **Smoke** |  |  | 0.19 |
| No | 2,454 (56.4%) | 386 (50.9%) |  |
| Yes | 1,814 (43.6%) | 325 (49.1%) |  |
| **Alcohol use** |  |  | <0.001 |
| No | 971 (19.0%) | 212 (28.7%) |  |
| Yes | 2,864 (81.0%) | 407 (71.3%) |  |
| **Hyperlipidaemia** |  |  | 0.683 |
| No | 2,640 (63.5%) | 450 (62.5%) |  |
| Yes | 1,610 (36.5%) | 257 (37.5%) |  |
| **Diabetes** |  |  | 0.001 |
| No | 3,761 (91.6%) | 577 (85.8%) |  |
| Yes | 511 (8.4%) | 134 (14.2%) |  |
| **Hypertension** |  |  | 0.11 |
| No | 2,573 (64.5%) | 411 (59.9%) |  |
| Yes | 1,699 (35.5%) | 300 (40.1%) |  |
| **CHD** |  |  | 0.062 |
| No | 4,106 (97.0%) | 690 (98.2%) |  |
| Yes | 153 (3.0%) | 19 (1.8%) |  |
| **Platelet** | 231 (197, 271) | 229 (195, 265) | 0.492 |
| **Monocyte** | 0.50 (0.40, 0.60) | 0.50 (0.40, 0.60) | 0.651 |
| **Lymphocyte** | 1.90 (1.60, 2.40) | 2.00 (1.70, 2.60) | 0.008 |
| **Neutrophils percent** | 60 (53, 66) | 57 (51, 63) | <0.001 |
| **Neutrophils** | 3.90 (3.00, 5.10) | 3.80 (3.10, 4.82) | 0.178 |
| **SIRI** | 1.00 (0.68, 1.47) | 0.89 (0.63, 1.26) | 0.003 |
| **SIRI tertile** |  |  | 0.180 |
| T1 | 1,393 (28.2%) | 265 (33.0%) |  |
| T2 | 1,402 (35.7%) | 245 (36.3%) |  |
| T3 | 1,477 (36.1%) | 201 (30.7%) |  |

BMI= body mass index; CHD= Coronary heart disease; IQR=interquartile range; PIR= family poverty to income ratio; SIRI=systemic inflammation response index.Continuous variables are presented as median (IQR); Categorical variables are presented as n (unweighted) (%);Wilcoxon rank-sum test for complex survey samples;chi-squared test with Rao & Scott's second-order correction.

**Supplementary Table 3. Weighted multivariable logistic regression for association between SIRI and LTBI.**

| **Variable** | **Molder1** | |  | **Molder2** | |  | **Molder3** | |  |
| --- | --- | --- | --- | --- | --- | --- | --- | --- | --- |
|  | **OR** | **95% CI** | ***p*-value** | **OR** | **95% CI** | ***p*-value** | **OR** | **95% CI** | ***p*-value** |
| **SIRI** | 0.72 | 0.61, 0.86 | 0.001 | 0.66 | 0.53, 0.81 | <0.001 | 0.80 | 0.67, 0.96 | 0.020 |
| **SIRI tertile** |  |  |  |  |  |  |  |  |  |
| T1(<0.73) | Ref. |  |  | Ref. |  |  | Ref. |  |  |
| T2(0.73-1.2) | 0.87 | 0.58, 1.31 | 0.474 | 0.83 | 0.55, 1.25 | 0.341 | 1.18 | 0.77, 1.82 | 0.425 |
| T3(>1.2) | 0.73 | 0.55, 0.96 | 0.025 | 0.63 | 0.47, 0.85 | 0.005 | 0.91 | 0.67, 1.22 | 0.494 |

Reference (Ref.); OR=Odds Ratio;CI= Confidence Interval; Molder1:unadjusted; Molder2: adjusted for gender and age; Molder 3:adjusted for gender, age, race, education levels, marital status, PIR, BMI, smoke, alcohol use, hypertension, diabetes, hyperlipidaemia, and CHD.

**Supplementary Table 4. Univariate analysis of immune cell parameters in blood tests related to LTBI.**

| **Characteristic** | **Total (n = 4983)** | **Non-LTBI**（N=4272) | **LTBI(N=711)** | ***P*** value |
| --- | --- | --- | --- | --- |
| White blood cell （10³ cells/μL） | 6.94 ± 2.24 | 6.97 ± 2.29 | 6.78 ± 1.94 | 0.034 |
| Lymphocyte （10³ cells/μL） | 2.07 ± 1.07 | 2.06 ± 1.13 | 2.10 ± 0.64 | 0.361 |
| Monocyte （10³ cells/μL） | 0.51 ± 0.24 | 0.51 ± 0.25 | 0.49 ± 0.17 | 0.041 |
| Neutrophils （10³ cells/μL） | 4.12 ± 1.66 | 4.15 ± 1.69 | 3.93 ± 1.52 | <0.001 |
| Eosinophils (10³ cells/uL) | 0.20 ± 0.15 | 0.19 ± 0.15 | 0.20 ± 0.16 | 0.225 |
| Basophils (10³ cells/uL) | 0.04 ± 0.06 | 0.04 ± 0.06 | 0.04 ± 0.06 | 0.748 |
| Lymphocyte percent (%) | 30.61 ± 8.76 | 30.38 ± 8.83 | 32.01 ± 8.25 | <0.001 |
| Monocyte percent (%) | 7.56 ± 2.55 | 7.57 ± 2.61 | 7.48 ± 2.16 | 0.382 |
| Neutrophils percent (%) | 58.30 ± 9.88 | 58.54 ± 9.96 | 56.85 ± 9.27 | <0.001 |
| Eosinophils percent (%) | 2.87 ± 2.03 | 2.85 ± 2.02 | 2.96 ± 2.13 | 0.179 |
| Basophils percent (%) | 0.71 ± 0.62 | 0.71 ± 0.61 | 0.74 ± 0.69 | 0.220 |

Reference (Ref.); OR=Odds Ratio;CI= Confidence Interval; Molder1:unadjusted; Molder2: adjusted for gender and age; Molder 3:adjusted for gender, age, race, education levels, marital status, PIR, BMI, smoke, alcohol use, hypertension, diabetes, hyperlipidaemia, and CHD.

**Supplementary Table 5. Multivariable logistic regression for association between immune cell parameters and LTBI.**

| **Variables** | **Model1** |  |  | **Model2** |  |  | **Model3** |  |
| --- | --- | --- | --- | --- | --- | --- | --- | --- |
|  | **OR (95%CI)** | ***P*** value |  | **OR (95%CI)** | ***P*** value |  | **OR (95%CI)** | ***P*** value |
| Neutrophils | 0.92 (0.87 ~ 0.97) | <.001 |  | 0.92 (0.87 ~ 0.97) | 0.002 |  | 0.95 (0.89 ~ 1.01) | 0.100 |
| Monocyte | 0.60 (0.39 ~ 0.94) | 0.026 |  | 0.46 (0.29 ~ 0.73) | 0.001 |  | 0.58 (0.33 ~ 1.00) | 0.050 |
| Lymphocyte | 1.03 (0.97 ~ 1.09) | 0.371 |  | 1.04 (0.98 ~ 1.11) | 0.166 |  | 1.02 (0.94 ~ 1.10) | 0.685 |
| Eosinophils | 1.37 (0.82 ~ 2.28) | 0.225 |  | 1.12 (0.66 ~ 1.89) | 0.675 |  | 0.92 (0.48 ~ 1.76) | 0.795 |
| Basophils | 1.24 (0.33 ~ 4.59) | 0.748 |  | 1.28 (0.35 ~ 4.75) | 0.711 |  | 2.64 (0.56 ~ 12.38) | 0.219 |

Reference (Ref.); OR=Odds Ratio;CI= Confidence Interval; Molder1:unadjusted; Molder2: adjusted for gender and age; Molder 3:adjusted for gender, age, race, education levels, marital status, PIR, BMI, smoke, alcohol use, hypertension, diabetes, hyperlipidaemia, and CHD.

**Supplementary Table 6. Weighted Multivariate Cox proportional hazard models for association between SIRI and all-cause mortality.**

| **Variable** | **Molder1** | |  | **Molder2** | |  | **Molder3** | |  |
| --- | --- | --- | --- | --- | --- | --- | --- | --- | --- |
|  | **HR** | **95% CI** | ***p*-value** | **HR** | **95% CI** | ***p*-value** | **HR** | **95% CI** | ***p*-value** |
| **SIRI** | 1.32 | 1.21, 1.43 | <0.001 | 1.16 | 1.08, 1.24 | <0.001 | 1.18 | 1.10, 1.27 | <0.001 |
| **SIRI tertile** |  |  |  |  |  |  |  |  |  |
| T1(<0.73) | Ref. |  |  | Ref. |  |  | Ref. |  |  |
| T2(0.73-1.2) | 1.73 | 1.29, 2.30 | <0.001 | 1.41 | 1.00, 2.00 | 0.05 | 1.57 | 1.10, 2.24 | 0.013 |
| T3(>1.2) | 3.16 | 2.23, 4.47 | <0.001 | 1.74 | 1.20, 2.52 | 0.003 | 1.82 | 1.32, 2.51 | <0.001 |

Reference (Ref.); HR=Hazard Ratio; CI=Confidence Interval; Molder1:unadjusted; Molder2: adjusted for gender and age; Molder 3:adjusted for gender, age, race, education levels, marital status, PIR, BMI, smoke, alcohol use, hypertension, diabetes, hyperlipidaemia, and CHD.

**Supplementary Table 7. Weighted Multivariate Cox proportional hazard models for association between SIRI and all-cause mortality in LTBI patients.**

| **Variable** | **Molder1** | |  | **Molder2** | |  | **Molder3** | |  |
| --- | --- | --- | --- | --- | --- | --- | --- | --- | --- |
|  | **HR** | **95% CI** | ***p*-value** | **HR** | **95% CI** | ***p*-value** | **HR** | **95% CI** | ***p*-value** |
| **SIRI** | 1.74 | 1.44, 2.10 | <0.001 | 1.72 | 1.39, 2.14 | <0.001 | 1.98 | 1.35, 2.92 | <0.001 |
| **SIRI tertile** |  |  |  |  |  |  |  |  |  |
| T1(<0.73) | Ref. |  |  | Ref. |  |  | Ref. |  |  |
| T2(0.73-1.2) | 0.71 | 0.34, 1.47 | 0.358 | 0.57 | 0.23, 1.40 | 0.22 | 0.57 | 0.23, 1.40 | 0.22 |
| T3(>1.2) | 3.11 | 1.70, 5.67 | <0.001 | 1.83 | 0.88, 3.82 | 0.105 | 1.83 | 0.88, 3.82 | 0.105 |

Reference (Ref.); HR=Hazard Ratio; CI=Confidence Interval; Molder1:unadjusted; Molder2: adjusted for gender and age; Molder 3:adjusted for gender, age, race, education levels, marital status, PIR, BMI, smoke, alcohol use, hypertension, diabetes, hyperlipidaemia, and CHD.

**Supplementary Table 8. Weighted Multivariate Cox proportional hazard models for association between SIRI and all-cause mortality in non-LTBI patients.**

| **Variable** | **Molder1** | |  | **Molder2** | |  | **Molder3** | |  |
| --- | --- | --- | --- | --- | --- | --- | --- | --- | --- |
|  | **HR** | **95% CI** | ***p*-value** | **HR** | **95% CI** | ***p*-value** | **HR** | **95% CI** | ***p*-value** |
| **SIRI** | 1.31 | 1.21 , 1.43 | <0.001 | 1.15 | 1.08 , 1.24 | <0.001 | 1.17 | 1.10 , 1.26 | <0.001 |
| **SIRI tertile** |  |  |  |  |  |  |  |  |  |
| T1(<0.73) | Ref. |  |  | Ref. |  |  | Ref. |  |  |
| T2(0.73-1.2) | 1.82 | 1.12, 2.95 | 0.015 | 1.47 | 0.90 , 2.41 | 0.123 | 1.66 | 0.96 , 2.87 | 0.072 |
| T3(>1.2) | 3.20 | 2.14 , 4.79 | <0.001 | 1.77 | 1.20 , 2.62 | 0.004 | 1.74 | 1.07 , 2.83 | 0.026 |

Reference (Ref.); HR=Hazard Ratio; CI=Confidence Interval; Molder1:unadjusted; Molder2: adjusted for gender and age; Molder 3:adjusted for gender, age, race, education levels, marital status, PIR, BMI, smoke, alcohol use, hypertension, diabetes, hyperlipidaemia, and CHD.

**Supplementary Table 9. Multivariate Cox proportional hazard models for association between LTBI and all-cause mortality.**

| **Variable** | **Molder1** | |  | **Molder2** | |  | **Molder3** | |  |
| --- | --- | --- | --- | --- | --- | --- | --- | --- | --- |
|  | **HR** | **95% CI** | ***p*-value** | **HR** | **95% CI** | ***p*-value** | **HR** | **95% CI** | ***p*-value** |
| **LTBI** |  |  |  |  |  |  |  |  |  |
| No | Ref. |  |  | Ref. |  |  | Ref. |  |  |
| Yes | 1.14 | 0.90, 1.44 | 0.281 | 0.87 | 0.69 , 1.10 | 0.235 | 1.06 | 0.81, 1.40 | 0.662 |

Reference (Ref.); HR=Hazard Ratio; CI=Confidence Interval; Molder1:unadjusted; Molder2: adjusted for gender and age; Molder 3:adjusted for gender, age, race, education levels, marital status, PIR, BMI, smoke, alcohol use, hypertension, diabetes, hyperlipidaemia, and CHD.

**Supplementary Table 10. Associations of SIRI with all-cause mortality in all populations and Non-LTBI populations.**

| **Variable** | **Model1** | |  | **Model2** | |  | **Model3** | |
| --- | --- | --- | --- | --- | --- | --- | --- | --- |
|  | **HR(95%CI)** | ***P*-value** |  | **HR(95%CI)** | ***P*-value** |  | **HR(95%CI)** | ***P*-value** |
| **All participants** |  |  |  |  |  |  |  |  |
| SIRI | 1.31(1.27,1.35) | <0.001 |  | 1.17(1.13,1.22) | <0.001 |  | 1.17(1.11,1.23) | <0.001 |
| SIRI tertile |  |  |  |  |  |  |  |  |
| T1(<0.73) | Ref. |  |  | Ref. |  |  | Ref. |  |
| T2(0.73-1.2) | 1.24 (0.66, 1.60) | 0.099 |  | 1.09(0.84,1.40) | 0.528 |  | 1.14(0.86,1.52) | 0.369 |
| T3(>1.2) | 2.85 (2.28, 3.55) | <0.001 |  | 1.66(1.32,2.09) | <0.001 |  | 1.46( 1.12, 1.91) | 0.005 |
| **Non-LTBI participants** |  |  |  |  |  |  |  |  |
| SIRI | 1.31 (1.27,1.35) | <0.001 |  | 1.16 (1.12,1.21) | <0.001 |  | 1.17 (1.11,1.22) | <0.001 |
| SIRI tertile |  |  |  |  |  |  |  |  |
| T1(<0.73) | Ref. |  |  | Ref. |  |  | Ref. |  |
| T2(0.73-1.2) | 1.42 (1.07,1.89) | 0.016 |  | 1.22 (0.91 ~ 1.62) | 0.179 |  | 1.34 (0.98 ~ 1.83) | 0.070 |
| T3(>1.2) | 3.32 (2.58 ~ 4.28) | <0.001 |  | 1.88 (1.45 ~ 2.44) | <0.001 |  | 1.81 (1.34 ~ 2.44) | <0.001 |

Reference (Ref.); HR=Hazard Ratio; CI=Confidence Interval; Model1:unadjusted; Model2: adjusted for gender and age; Model 3:adjusted for gender, age, race, education levels, marital status, PIR, BMI, smoke, alcohol use, hypertension, diabetes, hyperlipidaemia, and CHD.

**Supplementary Table 11.** **Univariate analysis of baseline immune cell parameters and all-cause mortality in patients with LTBI.**

| **Characteristic** | **Total (n = 711)** | **Survival group（n=628)** | **Death group(n=83)** | ***P* value** |
| --- | --- | --- | --- | --- |
| White blood cell （10³ cells/μL） | 6.78 ± 1.94 | 6.82 ± 1.93 | 6.49 ± 2.00 | 0.151 |
| Lymphocyte （10³ cells/μL） | 2.10 ± 0.64 | 2.14 ± 0.63 | 1.87 ± 0.68 | <.001 |
| Monocyte （10³ cells/μL） | 0.49 ± 0.17 | 0.49 ± 0.17 | 0.53 ± 0.19 | 0.051 |
| Neutrophils （10³ cells/μL） | 3.93 ± 1.52 | 3.94 ± 1.51 | 3.84 ± 1.60 | 0.557 |
| Eosinophils (10³ cells/uL) | 0.20 ± 0.16 | 0.20 ± 0.16 | 0.21 ± 0.18 | 0.436 |
| Basophils (10³ cells/uL) | 0.04 ± 0.06 | 0.04 ± 0.07 | 0.03 ± 0.05 | 0.014 |
| Lymphocyte percent (%) | 32.01 ± 8.25 | 32.29 ± 8.04 | 29.91 ± 9.49 | 0.032 |
| Monocyte percent (%) | 7.48 ± 2.16 | 7.36 ± 2.13 | 8.34 ± 2.23 | <.001 |
| Neutrophils percent (%) | 56.85 ± 9.27 | 56.72 ± 9.12 | 57.84 ± 10.31 | 0.300 |
| Eosinophils percent (%) | 2.96 ± 2.13 | 2.91 ± 2.08 | 3.34 ± 2.44 | 0.081 |
| Basophils percent (%) | 0.74 ± 0.69 | 0.76 ± 0.71 | 0.60 ± 0.48 | 0.047 |

**Supplementary Table 12. Multivariate Cox proportional hazard models for association between immune cell parameters and all-cause mortality in patients with LTBI.**

| **Variables** | **Model1** |  |  | **Model2** |  |  | **Model3** |  |
| --- | --- | --- | --- | --- | --- | --- | --- | --- |
|  | **HR (95%CI)** | ***P*** value |  | **HR (95%CI)** | ***P*** value |  | **HR (95%CI)** | ***P*** value |
| Neutrophils | 0.96 (0.83, 1.11) | 0.570 |  | 1.01 (0.86, 1.17) | 0.944 |  | 0.98 (0.81, 1.18) | 0.813 |
| Monocyte | 3.14 (0.98, 10.04) | 0.054 |  | 2.21 (0.64, 7.56) | 0.208 |  | 1.89 (0.45, 7.85) | 0.381 |
| Lymphocyte | 0.47 (0.31, 0.70) | <.001 |  | 0.76 (0.52, 1.11) | 0.159 |  | 0.67 (0.42, 1.05) | 0.083 |
| Eosinophils | 1.61 (0.48, 5.41) | 0.445 |  | 0.94 (0.26, 3.43) | 0.924 |  | 1.51 (0.28, 8.15) | 0.630 |
| Basophils | 0.01 (0.00, 0.95) | 0.047 |  | 0.04 (0.00, 2.93) | 0.143 |  | 0.02 (0.00, 2.81) | 0.122 |

Reference (Ref.); HR=Hazard Ratio;CI= Confidence Interval; Molder1:unadjusted; Molder2: adjusted for gender and age; Molder 3:adjusted for gender, age, race, education levels, marital status, PIR, BMI, smoke, alcohol use, hypertension, diabetes, hyperlipidaemia, and CHD.


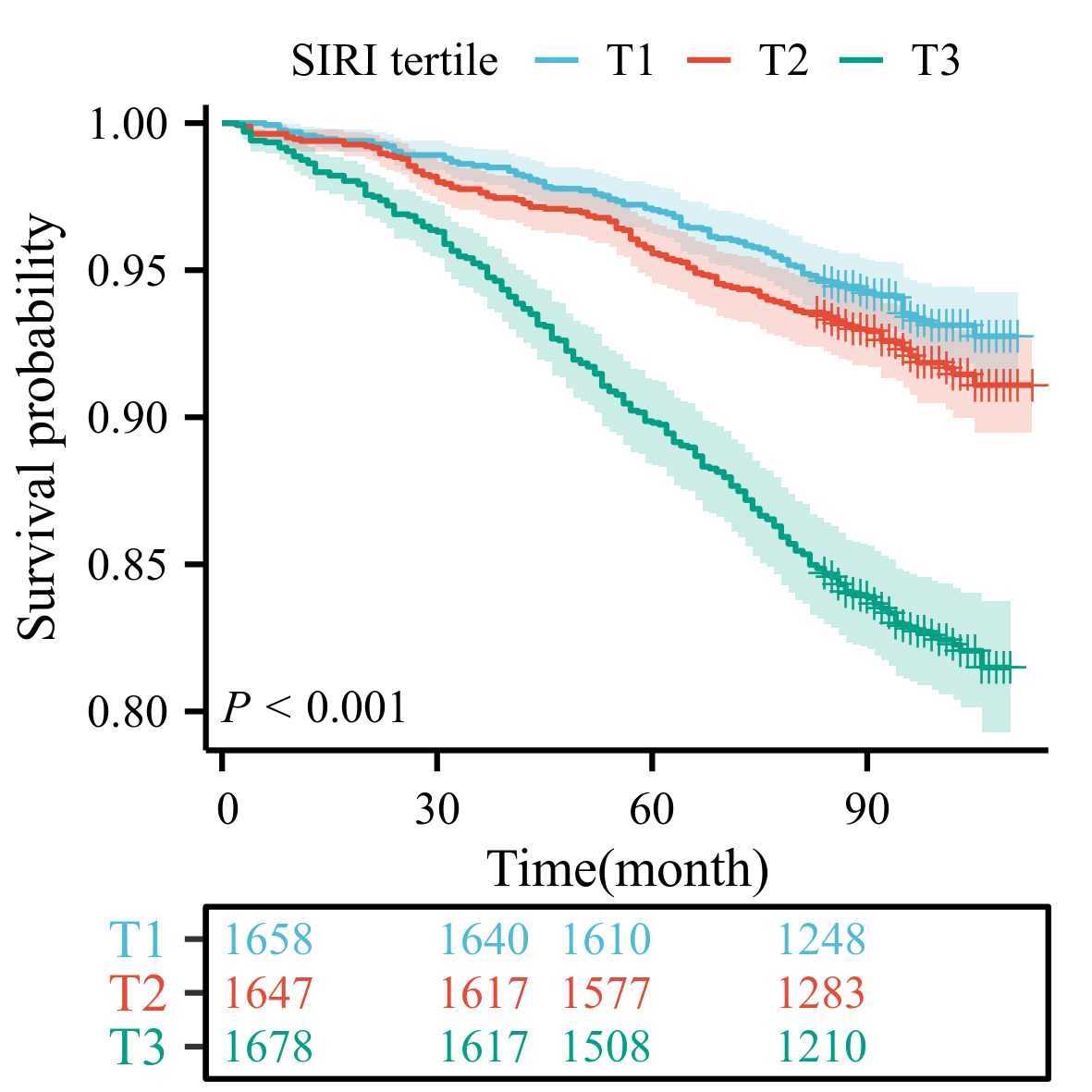


**Supplementary Figure 1. Kaplan-Meier survival curve for all-cause of all included participants.**


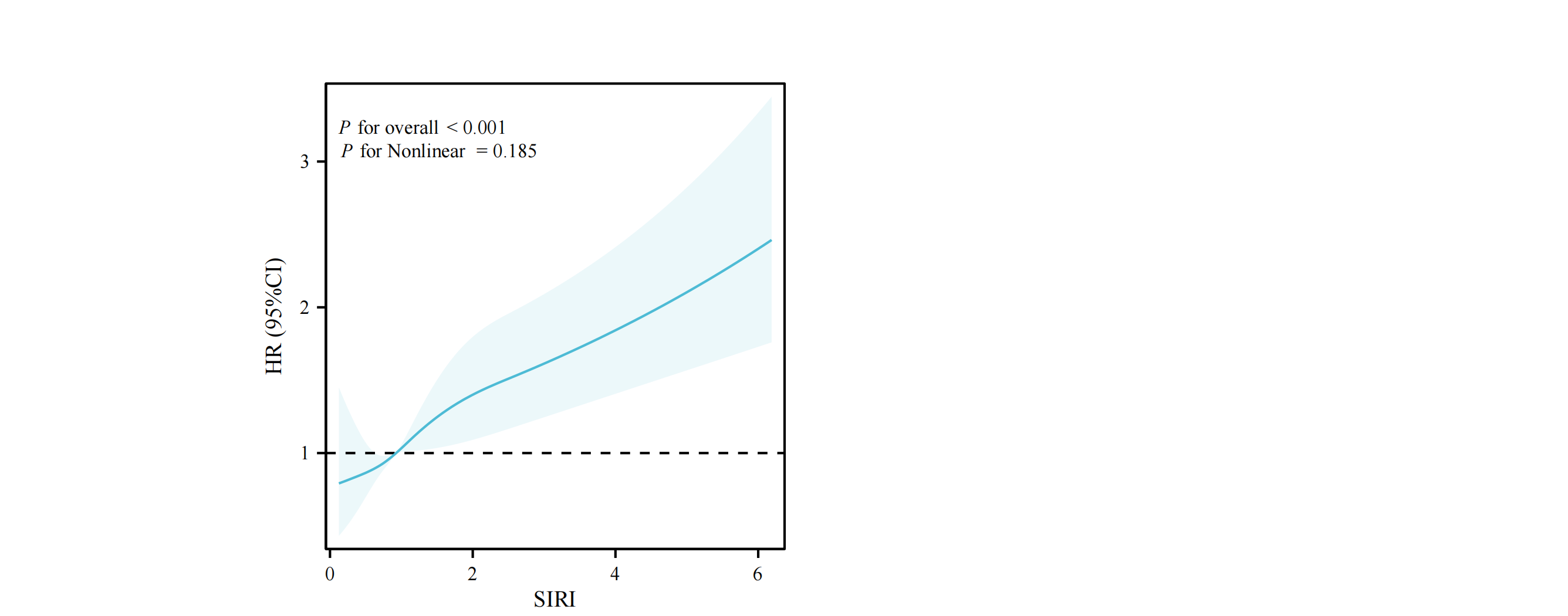


**Supplementary Figure 2. The association of SIRI with all-cause among all included participants visualized by restricted cubic spline.**


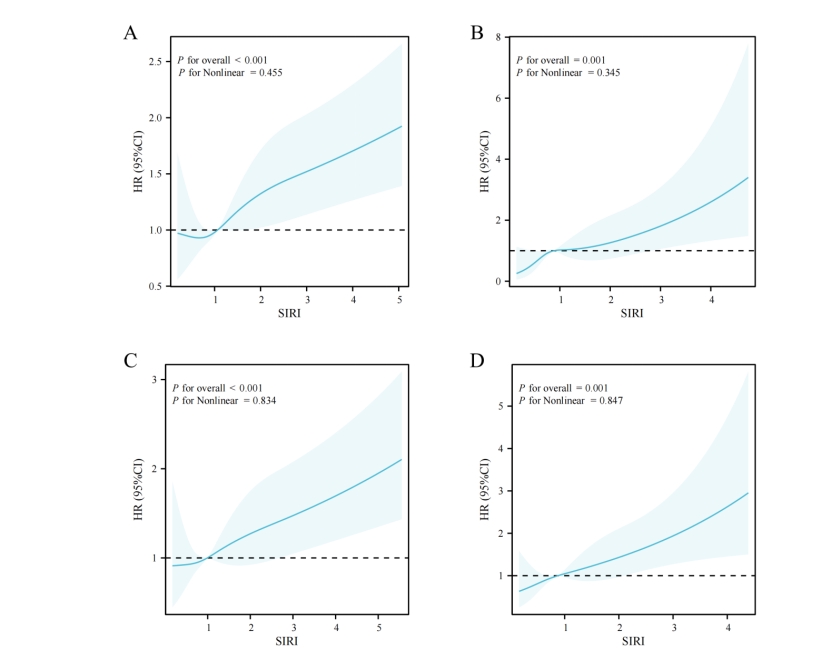


**Supplementary Figure 3. Restricted cubic spline analyses the association of SIRI with all-cause mortality in sex and age subgroups across all populations.** (A) Age<60 years,(B) Age ≥60years, (C) Gender=male, (D) Gender= Female. Adjusted for gender, age, race, education levels, marital status, PIR, BMI, smoke, alcohol use, hypertension, diabetes, hyperlipidaemia, and CHD. When analyzing gender groups, gender factors should be excluded from confounding factors, and the same applies when analyzing age subgroups.


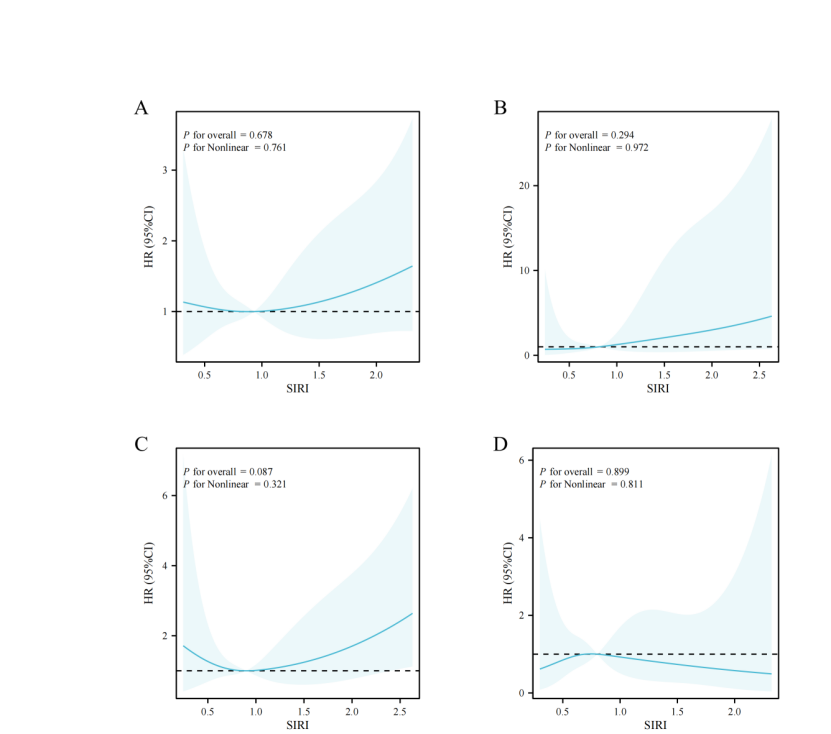


**Supplementary Figure 4. Restricted cubic spline analyses the association of SIRI with all-cause mortality in sex and age subgroups with LTBI populations.** (A) Age<60 years,(B) Age ≥60years, (C) Gender=male, (D) Gender= Female. Adjusted for gender, age, race, education levels, marital status, PIR, BMI, smoke, alcohol use, hypertension, diabetes, hyperlipidaemia, and CHD. When analyzing gender groups, gender factors should be excluded from confounding factors, and the same applies when analyzing age subgroups.


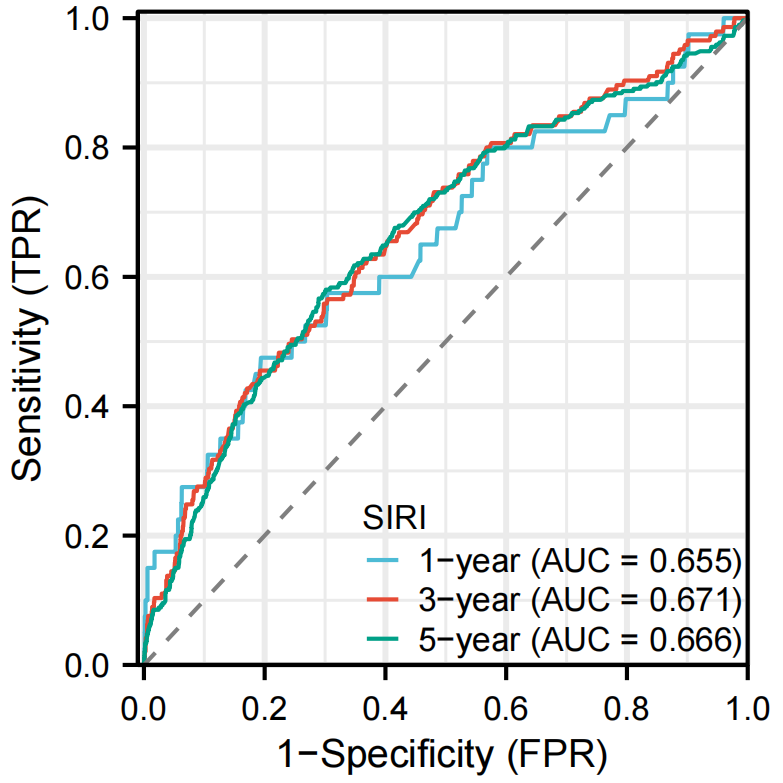


**Supplementary Figure 5. Time-dependent ROC curves of the SIRI for predicting all-cause mortality among all included participants.**


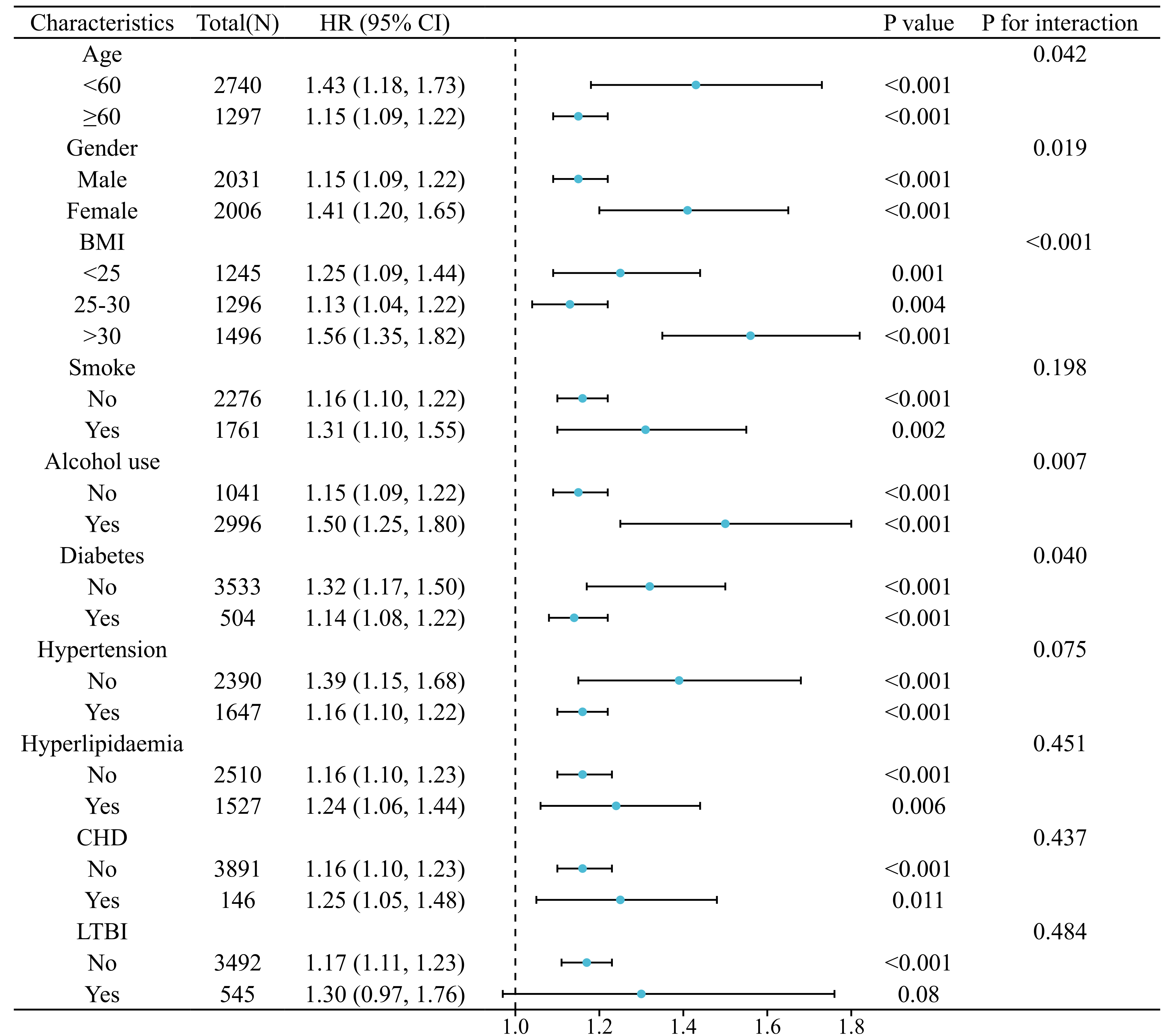


**Supplementary Figure 6.** **Stratified analysis of relationship between SIRI and risk of all-cause mortality in all patients.** (Adjusted for gender, age, race, education levels, marital status, PIR, BMI, smoke, alcohol use, hypertension, diabetes, hyperlipidaemia, and CHD.)
